# The therapeutic effect to eldecalcitol + bisphosphonate is superior to bisphosphonate alone in the treatment of osteoporosis: a meta-analysis

**DOI:** 10.1186/s13018-020-01896-z

**Published:** 2020-09-09

**Authors:** Zaoqian Zheng, Jinyu Luo

**Affiliations:** 1 Department of Pharmacy, Tongde Hospital of Zhejiang Province, Hangzhou, 310012 Zhejiang China; 2 Division of Medical Administration, Tongde Hospital of Zhejiang Province, Hangzhou, 310012 Zhejiang China; 3 Department of Medicine, Zhejiang Academy of Traditional Chinese Medicine, Hangzhou, 310012 Zhejiang China; 4 Hemopurification Center, Division of Nursing, Tongde Hospital of Zhejiang Province, No. 234 Gucui Road, Xihu District, Hangzhou, 310012 Zhejiang Province China

**Keywords:** Osteoporosis, Bisphosphonate, Eldecalcitol, Therapeutic effect, Bone mineral density, Meta-analysis

## Abstract

**Background:**

Osteoporosis is a metabolic bone disease. Bisphosphonate (BP) and eldecalcitol (ELD) are two clinical first-line drugs for osteoporosis patients. However, the effect of ELD + BP vs. BP alone on osteoporosis treatment is still unclear. The present meta-analysis was conducted to evaluate the different therapeutic effect of BP + ELD vs. BP alone in osteoporosis treatment.

**Methods:**

Eligible documents that selected from online databases including PubMed, Embase, and Cochrane Library were included in this study (updated to March 3, 2020). The quality assessment of the included studies was based on the guidelines of Cochrane. Meta-analysis was performed according to criteria such as intervention plan and outcome. The indicators including bone mineral density (BMD) in all enrolled studies were included in the current analysis. Pooled odds ratios (ORs) and weighted mean differences (WMDs) with 95% confidence intervals (CI) were calculated using fixed- or random-effects models. Then, heterogeneity analysis was performed based on Cochran’s *Q* test and *I*^2^ statistics.

**Results:**

A total of 4 studies (456 cases) with high quality were enrolled in this study. The effect of ELD + BP was superior to BP alone based on indicators including femoral neck BMD (FN-BMD) and total hip BMD (TH-BMD) in patients with followed up ≤ 6 months. Moreover, the effect of ELD + BP was superior to BP alone based on lumbar spine BMD (LS-BMD) in patients with 12 months followed up.

**Conclusion:**

Therapeutic effect of ELD + BP was superior to BP alone in osteoporotic patients based on the influence of BMD.

## Background

Osteoporosis is a group of bone diseases characterized by bone pain and easy fracture [1]. This disease lead to a total of 8.9 million fractures in 1 year all over the world [2]. Although the monitoring therapy based on bone mineral density (BMD) measurement contributes to the fracture diagnose, there is still a high mortality in people with osteoporosis due to the complications of fracture [3, 4]. Thus, effective drug prevention and treatment is necessary for people with osteoporosis or suffering osteoporotic fracture [5].

Bisphosphonate (BP) is one of the most commonly used first-line drugs for the clinical therapy of osteoporosis [6]. It contributes to the decrease of future fracture occurrence in patients with osteoporosis via change in the expression of blood mRNA in the process of osteoporosis [7, 8]. In Japan, a previous 2-year multicenter study shows that weekly used BP treatment significantly increases the quality of life osteoporosis patients [9]. On the contrary, some studies prove that BP not only is unsuitable for secondary osteoporosis treatment in children, but also do not appear to change the overall risk of death [10, 11]. Furthermore, eldecalcitol (ELD) is an active analog of vitamin D commonly used for the clinical treatment of osteoporosis [12]. A clinical study shows that ELD can reduce the re-absorption of blood calcium into the bone, improve the absorption of calcium in the intestine, and then further increase the bone density in osteoporosis patients [13]. Compared with other drugs such as alfacalcidol, ELD is more effective in preventing vertebral and wrist fractures in osteoporotic patients [14]. Actually, ELD + BP is useful for the osteoporotic patients who undergo the long-time BP therapy [15]. Increase in BMD by BP + ELD-treated osteoporotic patients is associated with the serum calcium level within the reference interval [16]. However, some studies indicated that compared with the BP-associated combination therapy, the BP treatment alone significantly increased the therapy effect in osteoporotic patients [17–19]. The clinical advantages of BP alone in the treatment of osteonecrosis compared with combined therapy have also been confirmed by multi-center studies [20]. To sum up, although previous studies prove the effect of BP alone or BP + ELD in clinical treatment of osteoporosis patients, it is still not clear which treatment strategy is better due to the limited sample size in each study. Therefore, it is necessary to explore more comprehensively and objectively the therapeutic effect of BP alone and BP + ELD on osteoporosis patients based on a meta-analysis.

In this study, the related articles which meet the certain inclusion criteria in Embase, PubMed, and Cochrane Library databases were searched. The indicators including BMD, blood calcium, blood phosphorus, and TRACP-5b in all enrolled RCT and non-RCT studies were included in the current analysis. Meta-analysis was performed according to criteria such as different indicators and outcome. The current study was aimed to evaluate the therapeutic effect of BP + ELD vs. BP in osteoporosis treatment.

## Methods

### Data sources

Relevant studies were searched from electronic databases including Embase, PubMed, and Cochrane Library (updated to March 3, 2020). The main searching keywords included the following: (“Eldecalcitol” OR “Bisphosphonate”) AND (“alendronate” OR “pamidronate” OR “ibandronate” OR “risedronate” OR “clodronate” OR “minodronate” OR “osteoporosis”). The detail information for search steps and corresponding results were shown in Table 1. Additionally, references of paper documents were hand searched for additional information on the procedures. No language restrictions were imposed in the current meta-analysis.

**Table 1:** The detail information for search steps and corresponding results (retrieval time: March 3, 2020)

Search	Query	Items found
#1	“eldecalcitol”[Supplementary Concept] OR “eldecalcitol”[All Fields]	177
#2	(“diphosphonates”[MeSH Terms] OR “diphosphonates”[All Fields] OR “bisphosphonate”[All Fields]) OR “diphosphonate”[All Fields]) OR “bisphosphonates”[All Fields]) OR Bisphosphate[All Fields] OR (“alendronate”[MeSH Terms] OR “alendronate”[All Fields]) OR (“pamidronate”[MeSH Terms] OR “pamidronate”[All Fields]) OR “zoledronic acid”[All Fields] OR (“ibandronic acid”[MeSH Terms] OR (“ibandronic”[All Fields] AND “acid”[All Fields]) OR “ibandronic acid”[All Fields] OR “ibandronate”[All Fields]) OR (“risedronic acid”[MeSH Terms] OR (“risedronic”[All Fields] AND “acid”[All Fields]) OR “risedronic acid”[All Fields] OR “risedronate”[All Fields]) OR (“clodronic acid”[MeSH Terms] OR (“clodronic”[All Fields] AND “acid”[All Fields]) OR “clodronic acid”[All Fields] OR “clodronate”[All Fields]) OR (“YM 529”[Supplementary Concept] OR “YM 529”[All Fields] OR “minodronate”[All Fields]) OR (“3-(2,2,2-trimethylhydrazine)propionate”[Supplementary Concept] OR “3-(2,2,2-trimethylhydrazine)propionate”[All Fields] OR “mildronate”[All Fields])	57,670
#3	“osteoporosis, postmenopausal”[MeSH Terms] OR (“osteoporosis”[All Fields] AND “postmenopausal”[All Fields]) OR “postmenopausal osteoporosis”[All Fields] OR “osteoporosis”[All Fields] OR “osteoporosis”[MeSH Terms]	87,049
#4	#1 AND #2 AND 3	33

### Inclusion and exclusion criteria

The inclusion criteria for articles were as follows: (i) the study subjects were patients with osteoporosis; (ii) the experimental group received ELD combined with BP treatment, and the control group was treated with BP alone (the types of BP (alendronate, ibandronate, risedronate, minodronate, etc.); (iii) the outcome of the study was based on indicators such as BMD (lumbar spine BMD (LS-BMD)), total hip BMD (TH-BMD), FN-BMD, blood calcium, blood phosphorus, and TRACP-5b; and (iv) research type was clinical research including RCT, non-RCT, and other clinical studies. The before-after studies were excluded from the analysis. If more than one study were published by the same sample, then only one study (the latest study or the completest study) was extracted. Moreover, the non-treatise literatures including reviews, letters, and comments were excluded.

### Data extraction and quality assessment

A total of 2 independent researchers participate in the data extraction in current meta-analysis. The strategies including discussion and reexamination were used for the problems during the article research. The available data including name of first author, year of publication, country, type of research, age, gender, sample size, and intervention plan, as well as outcome information in each literature, were enrolled in this study. Cochrane was used for the quality assessment [21]. A panel discussion with a third member was used to deal with disputes arising from current data retrieval process.

### Statistical analysis

The RevMan software (version: 5.3, Oxford, UK) was used for the statistical analysis in current study. Weighted mean difference (WMD) and 95% confidence interval (CI) were used as the effect size for the evaluation of continuous variable. The mean difference (MD) and standard deviation (SD) of each evaluation index before and after the intervention in the ELD + BP group and BP group were used as the effect values for difference comparison. Then, the meta-analysis was used to assess whether there was a significant difference in MD between the two groups.

Then, heterogeneity analysis was performed based on Cochran’s *Q* test and *I*^2^ statistics [22]. A random-effects model was used if heterogeneity was observed (*P* < 0.05 or *I*^2^ > 50%); otherwise, a fixed-effects model was applied. Furthermore, sensitivity analysis is used in the current research to analyze the impact of the combined outcomes after the literature was proposed one by one. Finally, the publication bias was evaluated by a funnel plot.

## Results

### Included studies

**Fig. 1:**
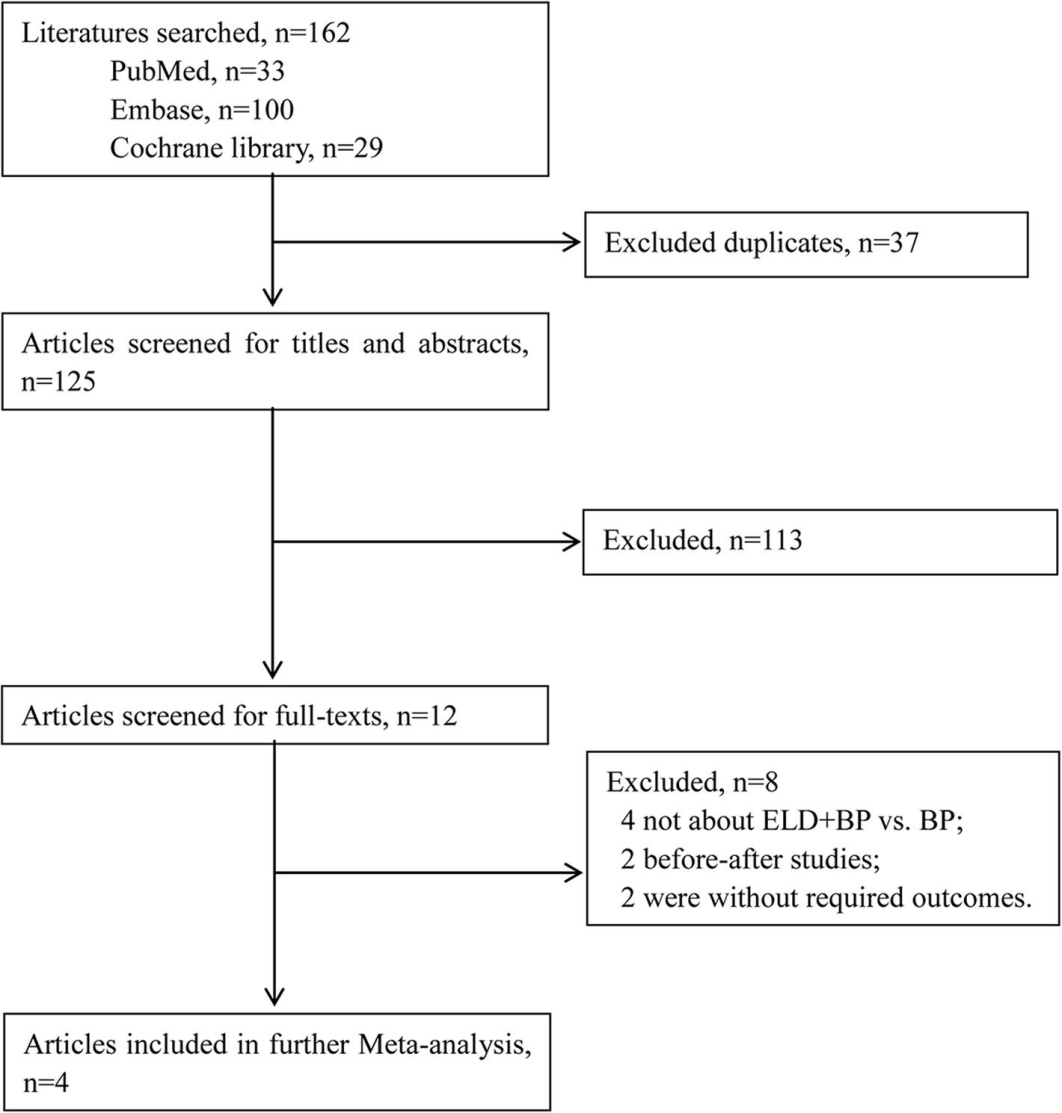
Flow diagram of screening process for eligible articles

A total of 33, 100, and 29 studies in PubMed, Embase, and Cochrane library database, respectively, were included (Fig. 1). After deletion of the duplicate articles, a total of 125 studies were selected. After reading the title and abstract, a total of 113 articles were further excluded because these studies were not eligible for inclusion criteria. From the remaining 12 studies, 8 studies (4 studies without ELD + BP vs. BP; 2 before-after studies and 2 studies without required outcomes) were filtered out after reading the full text. Finally, totally, 4 studies with sufficient data including 2 RCT [23, 24] and 2 non-RCT [25, 26] were enrolled for the present meta-analysis (Table 2).

**Table 2:** Characteristics of 4 included studies in this meta-analysis

Study	Area	Design	Duration	Criteria for osteoporosis	Duration	Group	Intervention	*n*	Age, years	Women, *n* (%)	BMI
Ebina et al. [28]	Japan	Non-RCT	NR	JSBMR	12 months	BP + ELD	MIN 50 mg/month, ELD 0.75 μg/day	80	71.8 ± 0.9	74 (92.5%)	21.3 ± 0.6
						BP	MIN 50 mg/month	63	71.7 ± 1.6	58 (92.1%)	21.5 ± 0.6
Iwamoto and Sato [23]	Japan	RCT	April 2012–July 2012	JDC	6 months	BP + ELD	ALE 35 mg weekly or risedronate 17.5 mg weekly, ELD 0.75 μg/day	50	72.3 ± 8.3	50 (100%)	22.5 ± 3.3
						BP	ALE 35 mg weekly or risedronate 17.5 mg weekly	46	69.1 ± 9.9	46 (100%)	22.7 ± 2.5
Suzuki et al. [29]	Japan	RCT	May 2016–August 2017	JSBMR	18 months	BP + ELD	MIN 50 mg/month, ELD 0.75 μg/day	14	68.7 ± 3.1	14 (100%)	20.4 ± 0.7
						BP	MIN 50 mg/month	14	65.8 ± 4.0	14 (100%)	20.1 ± 0.5
Takeuchi et al. [27]	Japan	Non-RCT	March 2014–January 2017	JDC	12 months	BP + ELD	Ibandronate 1 mg injections, ELD NR	94	NR	NR	NR
						BP	Ibandronate 1 mg injections	95	NR	NR	NR

*ALE* alendronate, *MIN* minodronate, *ELD* eldecalcitol, *BMI* body mass index, *BP* bisphosphonate, *JDC* Japanese diagnostic criteria, *JSBMR* Japanese Society for Bone and Mineral Research, *NR* not reported, *RCT* randomized controlled trial

### Information for all enrolled literatures

**Fig. 2:**
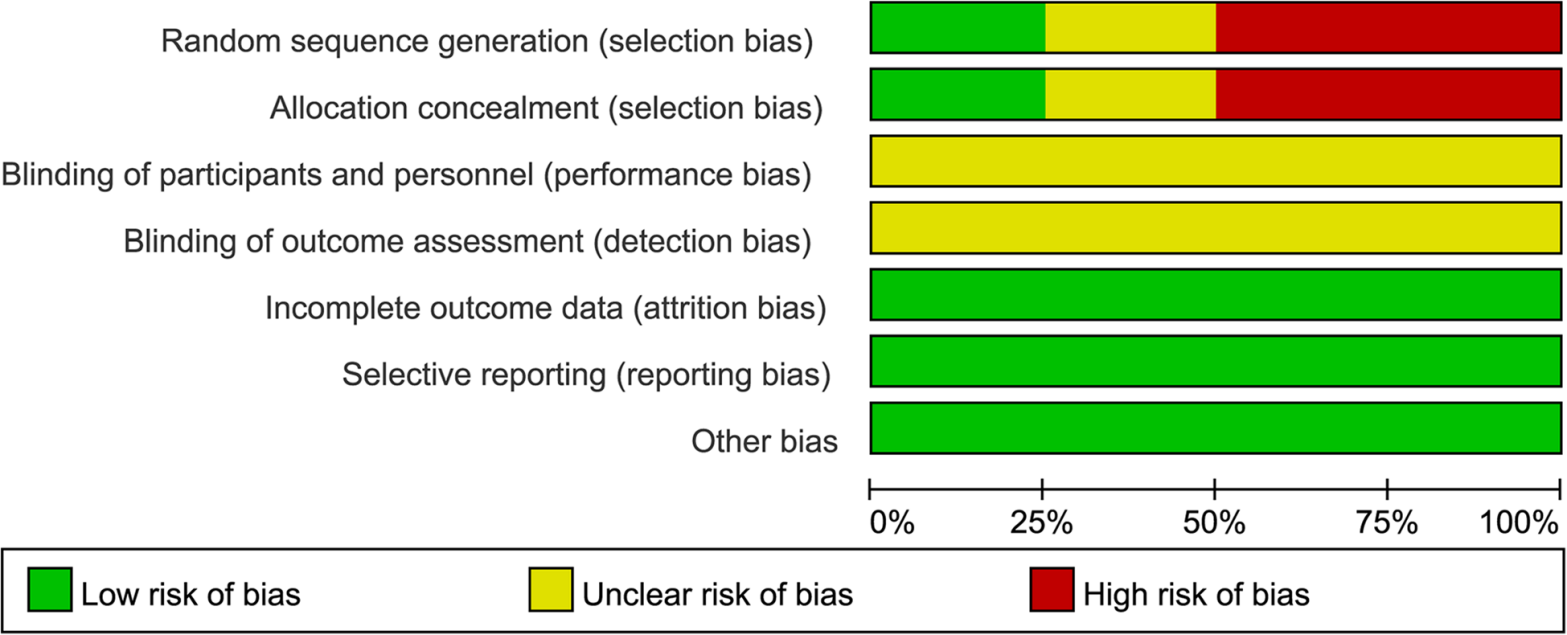
The publication bias evaluation based on enrolled literatures

**Fig. 3:**
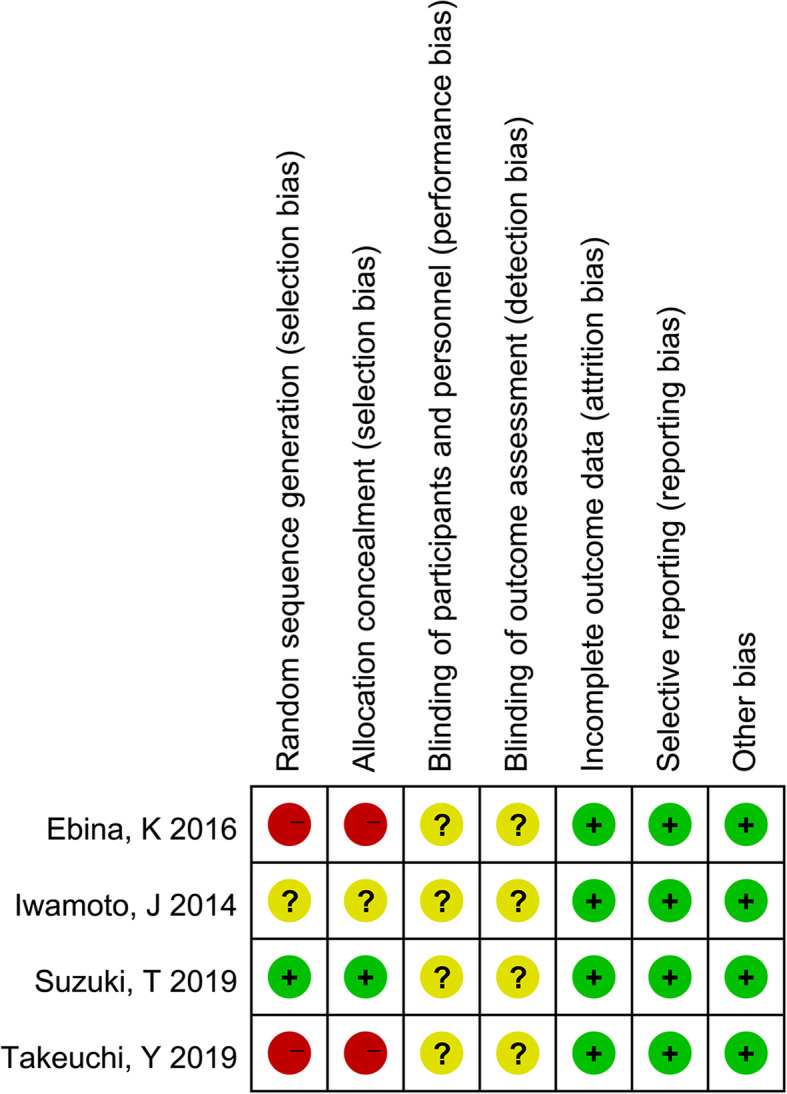
Summary of the risk of bias for each included study. “+” (green): low risk of bias; “?” (yellow): unclear risk of bias; “−” (red): high risk of bias

The publication year of the 4 literatures was from 2014 to 2019. The research areas mainly focused on Japan. A total of 456 cases were enrolled, including 238 in the ELD + BP group and 218 in the BP group. Except for the research by Takeuchi et al. [27], there was no significant difference in age and BMI between ELD + BP group and BP group in all studies. The intervention plan of Ebina et al. [28] was same with Suzuki et al. [29]. The follow-up period of each study was 6–18 months. The results of literature quality evaluation showed that the risk of selection bias in the included studies was relatively high. All studies had not reported the implementation of a blind method on measurement of implementers, research objects, and outcomes. Therefore, the evaluations of “blinding of participants and personnel” and “blinding of outcomes assessment” were defined as “unclear risk” (Fig. 2). However, the other evaluation items were defined as “low risk.” Overall, the methodological bias included in each literature was moderate (Fig. 3).

### Meta-analysis

**Fig. 4:**
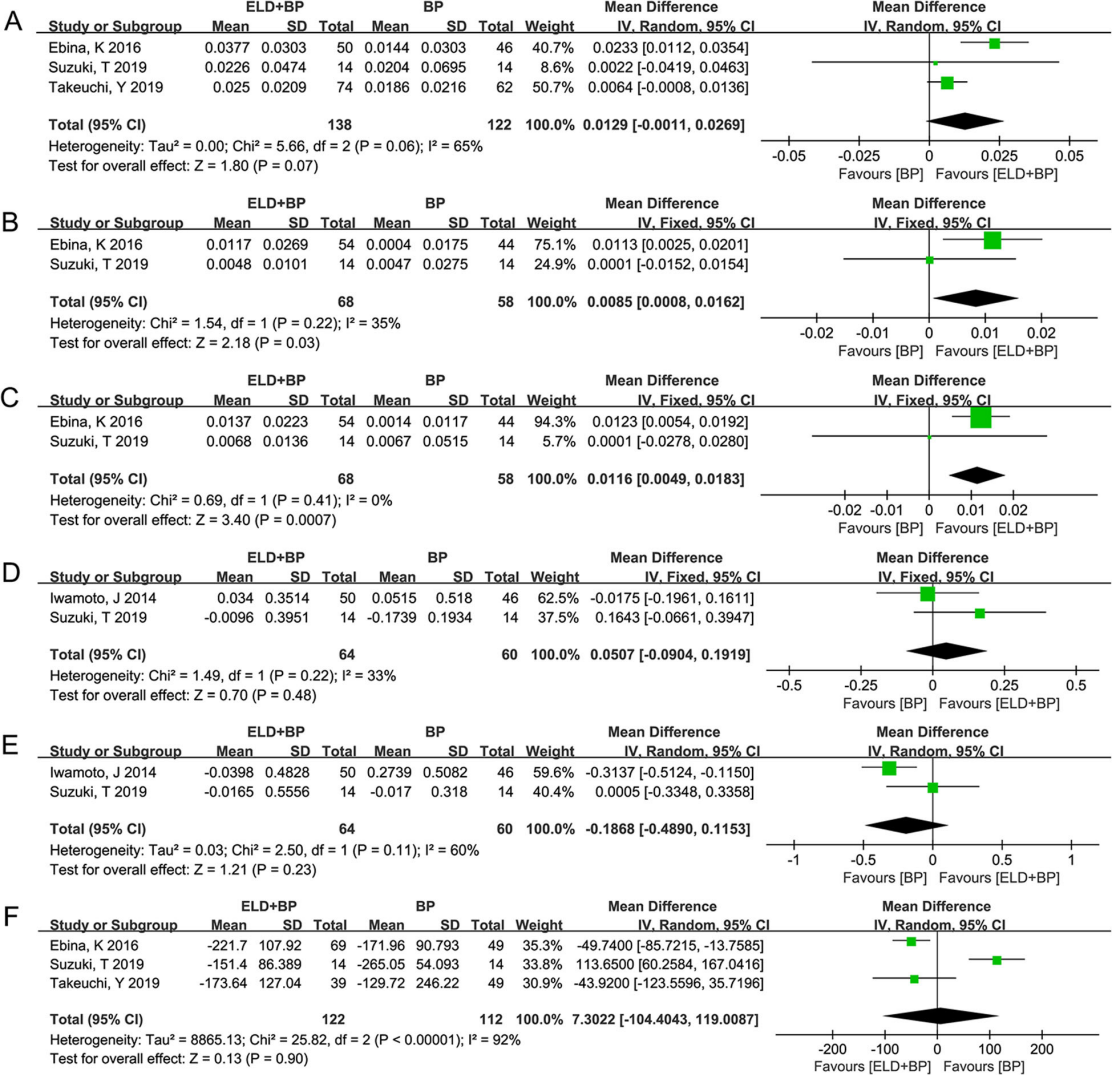
The results of meta-analysis for the effect of eldecalcitol (ELD) + bisphosphonate (BP) vs. BP on osteoporosis based on different indicators (intervention ≤ 6 months). **a** The pooled results from combing effect sizes for lumbar spine (LS) bone mineral density (BMD) using the random-effect model. **b**, **c** The pooled results from combing effect sizes for femoral neck (FN)-BMD and total hip (TH)-BMD using the fixed-effect model. **d**, **e** The meta-analysis for serum calcium and phosphorus. **f** The pooled results from combing effect sizes for serum TRACP-5b using the random-effect model

All events were divided according to the follow-up time (≤ 6 months and 12 months) in the subsequent analysis due to the different follow-up times in each study. A total of 3 studies [27–29] reported the outcomes of LS-BMD with follow-up time of less than 6 months. Since the heterogeneity test among these 3 studies [27–29] were statistically significant (*P* = 0.06, *I*^2^ = 65%), the random-effect model was used. The difference of LS-BMD between ELD + BP and BP (WMD (95%CI) = 0.0129 (− 0.0011, 0.0269) g/cm^2^, *P* = 0.07) (Fig. 4a) was not significant. Moreover, there were 2 studies [28, 29] that reported the outcomes of FN-BMD and TH-BMD with follow-up time less than 6 months. Since the heterogeneity test among these 2 studies [28, 29] were not statistically significant (*P* > 0.05, *I*^2^ < 50%), the fixed-effect model was used to calculate the WMD values and 95% CI. The difference between ELD + BP and BP for both FN-BMD (WMD (95%CI) = 0.0085 (0.0008, 0.0162) g/cm^2^) (Fig. 4b) and TH-BMD (WMD (95%CI) = 0.0116 (0.0049, 0.0183) g/cm^2^) (Fig. 4c) was significant, which further indicate the better effect of ELD + BP on increasing bone density. Furthermore, a total of 2 studies [29, 30] reported the outcomes of serum calcium and serum phosphorus with follow-up time less than 6 months. The results of meta-analysis for serum calcium (WMD (95% CI) = 0.0507 (− 0.0904, 0.1919) mg/dL, *P* = 0.48; heterogeneity: *I*^2^ = 33%, *P* = 0.22) and serum phosphorus (WMD (95%CI) = − 0.1868 (− 0.4890, 0.1153) mg/dL; heterogeneity: *I*^2^ = 60%, *P* = 0.23) showed that the difference of ELD + BP vs. BP was not significant in both serum calcium (Fig. 4d) and serum phosphorus (Fig. 4e). Additionally, a total of 3 studies [27–29] reported the outcomes of serum TRACP-5b with follow-up time less than 6 months. Since the heterogeneity test among these 3 studies [27–29] were statistically significant (*I*^2^ = 92%, *P* < 0.00001), the random-effect model was used. The difference of serum TRACP-5b between ELD + BP and BP (WMD (95%CI) = 7.3022 (− 104.4043, 119.0087) mU/dL, *P* = 0.90) (Fig. 4f) was not significant.

**Fig. 5:**
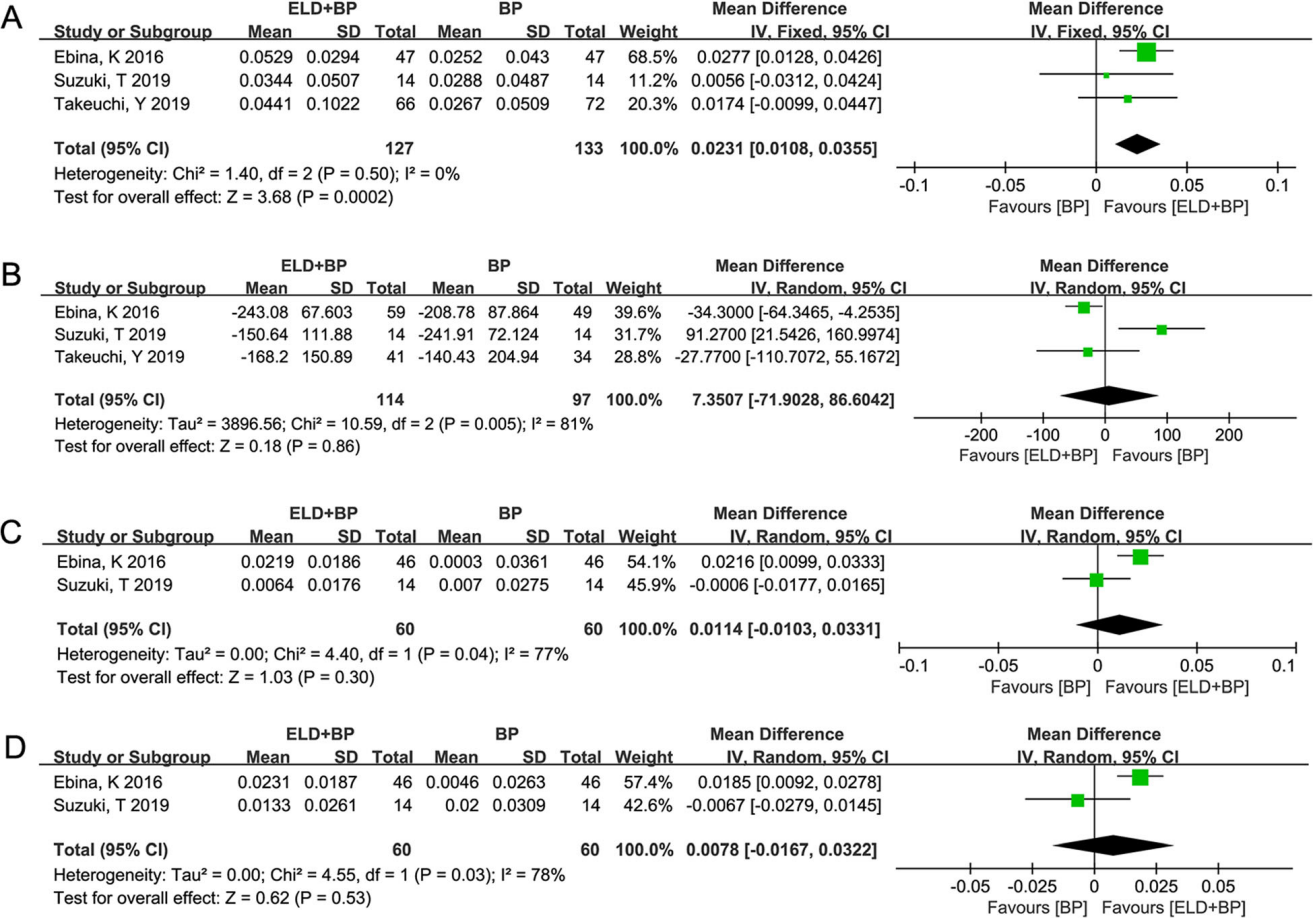
The results of meta-analysis for the effect of eldecalcitol (ELD) + bisphosphonate (BP) vs. BP on osteoporosis based on different indicators (intervention = 12 months). **a** The pooled results from combing effect sizes for lumbar spine (LS) bone mineral density (BMD) using the fixed-effect model. **b** The pooled results from combing effect sizes for serum TRACP-5b using the random-effect model. **c**, **d** The results of meta-analysis for femoral neck (FN)-BMD and total hip (TH)-BMD

Three studies [27–29] reported the results of LS-BMD (Fig. 5a) and serum TRACP-5b (Fig. 5b) with 12-month follow-up. The result showed that there was no significant heterogeneity (*I*^2^ = 0%, *P* = 0.50) of LS-BMD between ELD + BP and BP; thus, the fixed-effect model analysis was performed (WMD (95% CI) = 0.0231 (0.0108, 0.0355) g/cm^2^, *P* = 0.0002). Meanwhile, there was a significant heterogeneity (*I*^2^ = 81%, *P* = 0.005) of serum TRACP-5b between ELD + BP and BP, and the combined result of the random-effects model was WMD (95% CI) = 7.3507 (− 71.9028, 86.6042) mU/dL, *P* = 0.86. Moreover, two studies [28, 29] reported the comparison of FN-BMD (Fig. 5c) and TH-BMD (Fig. 5d) with 12-month follow-up. The result of the meta-analysis for FN-BMD was WMD (95% CI) = 0.0114 (− 0.0103, 0.0331) g/cm^2^, *P* = 0.30 (heterogeneity test result: *I*^2^ = 77%, *P* = 0.04). The result of TH-BMD was WMD (95% CI) = 0.0078 (− 0.0167, 0.0322) g/cm^2^, *P* = 0.53 (heterogeneity test result: *I*^2^ = 78%, *P* = 0.03). However, the meta-analysis was not performed on the outcomes of serum calcium and serum phosphorus with 12-month follow-up due to the small sample size (only 1 study reported).

## Discussion

The result of current meta-analysis showed that the effect of ELD + BP was superior to BP alone based on indicators including FN-BMD and TH-BMD in patients with followed up ≤ 6 months. Meanwhile, the effect of ELD + BP was superior to BP alone based on LS-BMD in patients with 12 months follow-up. However, there was no significant difference between ELD + BP and BP with other indicators such as serum calcium in patients with follow-up ≤ 6 months or 12 months.

The prevalence of osteoporosis and low bone mass can be estimated by FN-BMD and TH-BMD in adults 50 years and older [31, 32]. The quality of postmenopausal osteoporosis patients is increased when receiving weekly BP treatment [9]. Based on a certain mechanism of cell regulation, BP can improve the bone density of patients and reduce the bone turnover, so as to reduce the possibility of fracture [33]. However, elderly, postmenopausal, osteoporotic obese women are resistant to long-term BP, especially in regions of the TH, FN, and forearm compared with the spine [34]. Although BP is indicated in the prevention and treatment during the process of osteoporosis, BMD continues to decline in up to 15% of BP users [35]. It is proved that with the increase of time, BP can effectively slow down the speed of bone gain [36]. As an active analog of vitamin D, ELD is commonly used in the treatment of osteoporosis [12]. ELD can reduce the re-absorption of blood calcium into the bone, improve the absorption of calcium in the intestine, and then increase the bone density in osteoporosis patients [13]. A previous study shows that ELD has a significant effect in additionally decreasing the level of the bone resorption, even in the osteoporosis patients who undergo the long-time therapy of BP [37]. Actually, ELD + BP has been proven to be effective for the treatment of osteoporotic patients in Japan [38]. Kamimura et al. indicated that ELD provided additive increasing in patients with long-term BP therapy based on the detection of indicators such as BMD and bone turnover [15]. In this meat-analysis, the result showed that the effect of ELD + BP was superior to BP alone based on indicators including FN-BMD and TH-BMD in patients with follow-up ≤ 6 months. Meanwhile, the effect of ELD + BP was superior to BP alone based on LS-BMD in patients with 12 months follow-up. Thus, we speculated that the therapeutic effect of ELD + BP was superior to BP alone in osteoporotic patients based on the influence of BMD.

As we all know, the calcium balance or calcium homeostasis is very important to human bone health, which is achieved by the continuous transformation between osteoblasts and osteoclasts [39]. In postmenopausal women, ELD increases urinary calcium, consistent with an increase in intestinal absorption, and reduces markers of bone turnover [40]. Compared to alfacalcidol, ELD can effectively inhibit bone resorption by urinary serum calcium excretion, which further indicates a better osteoprotective effect of ELD in osteoporotic patients [41]. Furthermore, the effect of BP on bone resorption, calcium balance, and BMD has already been proven in vivo [42]. A previous study shows that BP can be used to regulate the serum calcium in hypercalcemia [43]. In the current meta-analysis, there was no significant difference between ELD + BP and BP with indicators such as serum calcium in patients with followed up ≤ 6 months or 12 months. This result showed that the difference in the effects of ELD + BP and BP alone on blood calcium regulation was not significant, indicating a good reliability of ELD + BP in clinical use.

There were some limitations in the current study. First, the heterogeneity in this study is not ideal. The reason for heterogeneity might be the types of drugs included in BP which were not identical. Second, there was no publication bias test for the current study, since there were only four studies included (only from Japan), which is not suitable for publication bias test. Even so, this study might be the first meta-analysis on the efficacy of ELD + BP vs. BP in the treatment of osteoporosis. Meanwhile, there was no significant heterogeneity in the included literature, indicating that the results of meta-analysis were reliable.

## Conclusion

In conclusion, the therapeutic effect of ELD + BP was superior to BP alone in osteoporotic patients based on the influence of BMD. However, further rigorous and high-quality validation studies based on a large sample size are needed in the future.

## Data Availability

The data used to support the findings of this study are available from the corresponding author upon request.
