# Correction to: The therapeutic effect to eldecalcitol + bisphosphonate is superior to bisphosphonate alone in the treatment of osteoporosis: a meta-analysis

**DOI:** 10.1186/s13018-020-01969-z

**Published:** 2020-09-21

**Authors:** Zaoqian Zheng, Jinyu Luo

**Affiliations:** 1 Department of Pharmacy, Tongde Hospital of Zhejiang Province, Hangzhou, 310012 Zhejiang China; 2 Division of Medical Administration, Tongde Hospital of Zhejiang Province, Hangzhou, 310012 Zhejiang China; 3 Department of Medicine, Zhejiang Academy of Traditional Chinese Medicine, Hangzhou, 310012 Zhejiang China; 4 Hemopurification Center, Division of Nursing, Tongde Hospital of Zhejiang Province, No. 234 Gucui Road, Xihu District, Hangzhou, 310012 Zhejiang Province China

**Correction to: Journal of Orthopaedic Surgery and Research 15, 390 (2020)**

**https://doi.org/10.1186/s13018-020-01896-z**

Following publication of the original article [1], the authors requested to do some changes to **Data sources**, part of **Methods** section as follows:

“The main searching keywords included the following: (“Eldecalcitol” OR “Bisphosphonate”) AND (“alendronate” OR “pamidronate” OR “ibandronate” OR “risedronate” OR “clodronate” OR “minodronate osteoporosis”).”

Should be changed to:

“The main searching keywords included the following: (“Eldecalcitol”) AND (“Bisphosphonate” OR “alendronate” OR “pamidronate” OR “ibandronate” OR “risedronate” OR “clodronate” OR “minodronate”) AND (“osteoporosis”).”
